# A Dog Is a Doctor's Best Friend: The Use of a Service Dog as a Perioperative Assistant

**DOI:** 10.1155/2016/9013520

**Published:** 2016-10-23

**Authors:** Shannon Tew, Brad M. Taicher

**Affiliations:** Duke University Medical Center, Durham, NC, USA

## Abstract

Service dogs are beneficial in providing assistance to people with multiple types of disabilities and medical disorders including visual impairment, physical disabilities, seizure disorders, diabetes, and mental illness. Some service animals have been trained as a screening tool for cancer. We review a case involving a 6-year-old female with a history of mast cell mediator release and immediate hypersensitivity due to the urticaria pigmentosa variant of cutaneous mastocytosis who underwent a cystourethroscopy. Her service dog, JJ, who would alert to mast cell mediator release, was used throughout the perioperative course as a means of anxiolysis and comfort and to monitor for mast cell mediator release. This case presents an example of a service dog used in a family-care model in the field of anesthesiology and provides a unique example of using a service dog as an additional monitor to alert the care team for impending mast cell mediator release.

## 1. Introduction

Service animals are widely used pertaining to disabilities or medical conditions, such as visual impairment, physical disabilities, seizure disorders, diabetes, and mental illness [1–5]. Some service animals have also been trained as a screening tool for cancer [6, 7]. In the practice of anesthesiology, the utility of service animals has rarely been reported despite recognized benefits in other health care settings. We describe a case involving a 6-year-old with history of mast cell mediator release due to the urticaria pigmentosa variant of cutaneous mastocytosis who underwent a cystourethroscopy. Her service dog, JJ, would alert her handler prior to clinical manifestations of mast cell mediator release. JJ was present throughout the perioperative period as a means of anxiolysis and comfort for the patient and to alert to mast cell mediator release. Written consent for photographs and publication was obtained from the patient's legal designee for protected health information.

## 2. Case Description

A 6-year-old 24-kilogram female presented for general anesthesia for a cystourethroscopy and a pressure induced cystogram with possible Deflux injection to evaluate and treat recurrent febrile urinary tract infections. She had a history of mast cell mediator release and immediate hypersensitivity due to the urticaria pigmentosa variant of cutaneous mastocytosis. Triggers of mast cell mediator release included abrupt environmental temperature changes, fever, exercise, stress, and fatigue. Due to frequent episodes of mast cell mediator release and hospitalizations, she was accompanied by a service dog, JJ, that was trained to alert when detecting olfactory molecules related to mast cell mediator release. JJ was rescued from an animal shelter when she was very young and is believed to be a terrier mix. She began 14 months of training to become a service animal and lived with the patient for 18 months leading up to the procedure. JJ was trained using operant conditioning with positive reinforcement to alert to specific stimuli. While she was initially trained to detect hyperglycemia and hypoglycemia, she was then trained to respond to scent samples from the clothing the patient was wearing when she had a significant reaction. Once she was reliably alerting to these samples in training, she was exposed to samples provided by two other patients with mastocytosis, and she reliably alerted to those too. JJ alerted to sensing minor reactions by circling behavior and more serious reactions by barking and tugging at the caretaker's clothing. Over the past 5 years, there were 3 known episodes where JJ failed to alert to a mild reaction, and all were during thunderstorms.

**Figure 1:**
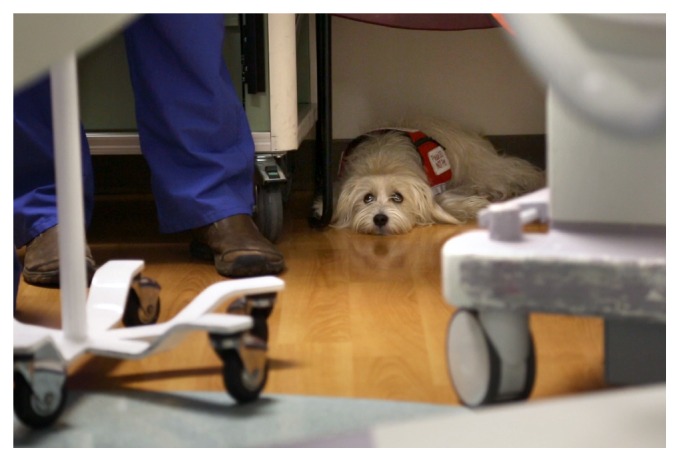
Service dog present in the procedure suite. She was present under the handler's chair near the anesthesia team and would alert to any perceived detection of mast cell degranulation.

Preoperatively, the patient was placed in an isolation room in the preoperative holding area. JJ was present as a form of comfort and anxiolysis to minimize stress, since this was a known trigger for her mast cell mediator release and immediate hypersensitivity. Other patients and families were alerted to the presence of the dog and screened for allergy to animal dander or other concerns. Since there are no reports of Deflux injection in a child with mastocytosis, there was heightened concern for intraoperative mast cell degranulation during injection. There was additional concern since the patient experienced significant cutaneous flushing during anesthetic emergence that required therapy following a previous anesthetic for an MRI. This procedure was clean-contaminated and occurred in a procedure suite, not an operating room. In conjunction with hospital infection control, the decision was made to bring JJ into the procedure suite as continued anxiolysis and as an additional monitor to alert the care team to potential evidence of mast cell mediator release. JJ was present under the handler's chair near the anesthesia team throughout the intraoperative period (Figure 1). We did not feel it was appropriate to have the patient's mother present throughout the procedure to handle the service animal, so JJ's trainer volunteered to serve the role as intraoperative handler and communicate with the anesthesia team about the dog's behavior.

ASA standard monitors were applied, and general anesthesia was induced with propofol 4 mg/kg and dexmedetomidine 0.5 *μ*g/kg IV. After loss of lash reflex, the patient was easily ventilated and a laryngeal mask airway (LMA) was placed. During this time, the handler communicated that JJ displayed circling behavior consistent with a minor reaction. There were no clinical signs of flushing or changes in vital signs. The patient was maintained on sevoflurane in air and oxygen and a dexmedetomidine infusion at 0.5 *μ*g/kg/hr. Injection of contrast was uneventful, and, due to no signs of vesicoureteral reflux, the injection of Deflux was not required. She received fentanyl 0.5 *μ*g/kg IV and received an additional bolus of dexmedetomidine 0.5 *μ*g/kg IV prior to emergence. After cystoscope removal, the LMA was removed under deep anesthesia. Shortly after this, the handler communicated that JJ displayed circling behavior consistent with a minor reaction, again with no clinical signs of flushing or changes in vital signs. The patient tolerated the procedure well, and she was then transported to the postanesthesia care unit (PACU) isolation room with her service dog. Thirty minutes into the PACU stay, JJ again exhibited circling behavior, alerting to a minor reaction that was not apparent by physical exam. There were no signs or symptoms of significant mast cell mediator release throughout the perioperative course, and there was no need for any alteration of the intended anesthetic plan.

## 3. Discussion 

In the field of anesthesiology, the development of family-centered care guidelines and policy statements has not been established, despite its presence in other medical fields [8]. Chorney and Kain suggest that certain factors, such as parental/patient anxiety, history with medical procedures, and coping styles, should be taken into account when establishing a perioperative plan for a pediatric patient [8]. The role of a service dog has been shown to be beneficial in a family-centered care model in the perioperative environment [9]. In this case, the dog provided perioperative anxiolysis and comfort for the child, decreasing the requirement of preoperative pharmacologic anxiolysis, and provided a more comfortable environment for the child. As she was at risk for mast cell degranulation, and stress was a known trigger, providing a calm and comforting experience for the patient was important in her psychological and medical management. Minimizing medication exposure, another potential trigger, was also important to decrease the potential risk for immediate hypersensitivity due to medications.

Although it is important to work with the medical team, family members, and patient to come to a consensus on an optimal management strategy, it is also important to consider adverse effects or unintended consequences. Given that a service dog was used throughout the perioperative period, the logistics, safety, and well-being of other patients needed to be considered. This included open communication with healthcare staff members, patients, and their families and infection control. All perioperative employees involved with the patient's care were aware of the service animal's presence and had no concerns about participating in her care. We also developed a plan with JJ's handler and circulating nurse that if the handler needed to exit or the dog became a distraction, the nurse would escort both out the back door, exiting the procedure suite.

Potential allergen exposure to other patients, their families, and staff members was a concern. We alerted all staff members, patients, and their family members to the service dog's presence and screened them for an allergy to animal dander. Despite our conservative approach, there are studies demonstrating that animal allergens have been present in locations where no animals reside, such as in schools and other public buildings [10, 11]. Factors that affect allergen transfer include clothing type and frequency of washing, human hair, and the number of people that have frequent contact with these animals [11]. We kept the patient and dog isolated from the other patients in the preoperative holding area and the PACU, and only the handler was in contact with the dog during the perioperative period. No concerns or adverse events were reported by other families or staff members.

Overall, our team felt that the benefits of having the service dog present outweighed the potential risks, and we minimized all risks present. As this was a clean-contaminated procedure, we elected that it takes place in a nonsterile procedure suite as opposed to an operating room to avoid contaminating a sterile environment. JJ remained on the floor and made contact only with her trainer throughout the perioperative period in an effort to minimize contact with linens, medical equipment, and personnel. JJ never came in contact with other patients and remained in isolated rooms throughout the perioperative course. Following JJ's exit from each perioperative room, the rooms were terminally cleaned prior to other patients entering.

Service dogs have been reported to detect melanoma, hypoglycemia, and seizures [3, 4, 6]. However, there is no documented report of a service dog used to detect mast cell mediator release in patients with mastocytosis. This service dog was used not only in a family-centered care model, but also as an additional perioperative monitor. During the cystourethroscopy, JJ's alerts only suggested less serious reactions, but these instances coincided with times in which a patient was most likely to be subject to increased stress, namely, anesthetic induction and emergence. However, the patient had no flushing or change in vital signs during any of these events. Although the mechanism is unknown, we hypothesize that JJ was able to alert to subclinical levels of mast cell mediator release coinciding with patient stress, a known trigger for our patient. Since JJ was trained via scent samples from the patient, we hypothesize that the mechanism involved in detection of mast cell mediator release includes molecules that the dog can detect via her olfactory system.

This case describes the use of a service dog in the perioperative period. The service dog was an integral part of creating a family-centered care model for this pediatric patient and also served uniquely as an additional monitor for mast cell degranulation.
